# Supplementary material for: An Innovative Scoring System to Select the Optimal Surgery in Breast Cancer after Neoadjuvant Chemotherapy
Source: J Pers Med. 2023 Aug 21;13(8):1280. doi: 10.3390/jpm13081280 (PMC10455579; doi:10.3390/jpm13081280)
Supplement: Supplementary file 1 [file jpm-13-01280-s001.zip › jpm-2504816-supplementary.pdf]

Journal of Personalized Medicine

MDPI

An Innovative Scoring System to select the optimal surgery in breast cancer after Neoadjuvant Chemotherapy

Antonio Franco [1,†], Alba Di Leone [1,†], Marco Conti [2], Alessandra Fabi [3,*], Luisa Carbognin [4], Andreina Daniela Terribile [1], Paolo Belli [2], Armando Orlandi [5], Martin Alejandro Sanchez [1], Francesca Moschella [1], Elena Jane Mason [1], Alessandra De Filippis [2], Giovanni Cimino [2], Fabio Marazzi [6], Ida Paris [4], Giuseppe Visconti [7], Lorenzo Scardina [1], Sabatino D'Archi [1], Marzia Salgarello [7], Diana Giannarelli [8], Riccardo Masetti [1] and Gianluca Franceschini [1]

Supplementary Materials:

Informations about patients and surgery indication:

1. Outpatient evaluation

Patients are assessed on an outpatient basis with evaluation of: Age at diagnosis; Menopausal state (fertile age, menopause); Body mass index (BMI); Current or previous history of cigarette smoking; Comorbidity; Previous history of both benign and malignant breast surgery and location of the cancer. The breast was divided into 5 quadrants: 1. Upper-outer quadrant (UOQ) also including the axillary extension; 2. Upper-internal quadrant (UIQ); 3. Lower-internal quadrant (LIQ); 4. Lower-outer quadrant (LOQ); 5. Retro-areolar quadrant (RA).

During the same visit, two photographs of the breasts with a temporary skin tattoo showing the cancer's projection and information regarding the degree of ptosis[1] and size of the bra are also obtained.

2 Histological definition

The histological definition was obtained by:

- Core Needle Biopsy (CNB) with evaluation of the tumor histotype (Invasive Ductal Carcinoma - DIC; Invasive Lobular Carcinoma - LIC; Invasive Carcinoma not classifiable in previous histotypes - CI NST), hormone receptors (Estrogen - ER; Progesterone - PgR; Androgen - AR) and prognostic factors (Human epidermal growth factor receptor 2 - HER2 and the proliferation index Ki-67). We have thus defined the tumor subtype: Luminal A (each ER≥1%; each PgR≥1%; Ki67 <20%; HER2 negative), Luminal B (each ER≥1%; each PgR≥1%; Ki67 ≥ 20%; HER2 negative), HER2 positive (HER2 +) (any ER, PgR and Ki67% with HER2 positive; or each ER≥1%; PgR <1%; each Ki67; HER2 negative) and triple negative (TN) (ER <1%; PgR <1%; any Ki67; HER2 negative). A non-magnetic clip was placed inside the neoplasm in the biopsy site in order to perform pre-surgical localization in case of a complete clinical response (cCR) or clinical regression to a non-palpable neoplasm. [2]

- CNB or Fine Needle aspiration cytology (FNAC) of the axilla in case of evidence of 1 to 3 suspected lymph nodes. Ultrasound criteria for suspicion of lymph node metastasis included: 1. diffuse cortical thickening (> 3mm), 2. eccentric cortical thickening, 3. focal cortical bulge, 4. diffusely hypoechoic rounded lymph node, 4. complete or partial disappearance of the adipose hilum, 5. cortical vascularization on color Doppler analysis, 6. complete or partial replacement of the lymph node with an ill-defined / irregular mass, 7. microcalcifications in the lymph node. In patients undergoing biopsy of the axillary lymph node(s), a non-magnetic clip was placed in the biopsied lymph node so that it could be located and subsequently removed during surgery.

3. Loco-regional and systemic staging.

Loco-regional and systemic staging was obtained by: Breast and axillary ultrasound (B-EUS; A-EUS); Mammography (RX) integrated with tomography; Contrast-enhanced magnetic resonance imaging (MRI); Total body CT scan (TB-CT) or Positron Emission Tomography (PET) scan; Bone scan

4. Radiological evaluation of imaging:

For the definition of the scoring system, MRI and RX have been revised with the aim of defining:

- Cancer extension: largest diameter measured on MRI
- Focality of breast lesions: divided into: 1. unifocal lesion: presence of a single breast lesion. 2. multifocal lesions: presence of two or more lesions located in the same quadrant or in two adjacent quadrants. 3. multicentric lesions: two or more breast lesions located in more than two quadrants or in quadrants distant from each other.
- Prediction of excised breast area on MRI (MRI-PEBA) [3]: Measurements of tumor volume (TV) and breast volume (BV) were performed by semi-automatic analysis using a dedicated GE Advantage Workstation 4.2 (GE Healthcare, Inc., Waukesha, WI, USA). The TV was calculated by plotting the maximum diameter of the neoplasm in the axial plane in the first dynamic post-contrast images, with the software which then automatically contoured the boundaries of the lesion, considering the voxels with an enhancement threshold greater than or equal to 70% compared to the signal intensity in the pre-contrast images, with subsequent manual adjustment of the edges to simulate the ideal volume to be removed (Figure S1). In the presence of multifocal or multicentric disease, the measurement included all suspected enhancement areas, including the area of healthy breast parenchyma between them, to simulate the volume that should ideally be removed by the surgeon. The VB measurement was calculated by tracing the breast edges every 1.5-2.0 cm on the dynamic axial images with the software which then completed the marking between the tracks. All measurements were repeated by two operators. MRI-PEBA was obtained with the formula: MRI-PEBA = TV/BV x 100
- Prediction of the excised breast area on RX (RX-PEBA)(4): Using the method introduced by Katariya, [5] we calculated the volume of the mammary gland considered as a cone ($\frac{1}{3}\pi r^2 h$) and evaluated the volume of glandular removed ($\frac{4}{3}(lesion\ length + 1\ cm)^3$). Finally, we estimated the percentage of excised breast volume using the ratio between the two previous formulas: $\frac{4(lesion\ length+1\ cm)^3}{r^2 h}$. For multifocal lesions, the evaluation of the volume of gland to be removed was calculated as a radius corresponding to the radius of the glandular parenchyma containing all the lesions. While in the case of multicentric disease we used the formula: 〖 $\frac{4\ \Sigma(length\ of\ n\ lesions\ +1\ cm)^3}{r^2 h}$ (Figure S1)
- Rancati score[6]: breast classification based on distance, detected on the mammogram X-ray, between the skin and the mammary gland.
- Extension of microcalcifications (maximum diameter) assessed on RX-mammography.

5. Neoadjuvant treatment

The most common therapeutic scheme administered consisted of Anthracycline plus Cyclophosphamide for 4 cycles every 3 weeks; followed by Docetaxel for 4 cycles every 3 weeks or weekly Paclitaxel for 12 cycles. In HER2+ patients, Trastuzumab was added for a total of 1 year. In patients with triple negative subtype cancer, Carboplatin could be added to weekly Paclitaxel for 12 cycles. [7-8]

6. Surgical indication

Patients included in the study underwent three types of surgery: BCS, OPS and CMR. Surgical planning was always discussed in a multidisciplinary dedicated surgery board. BCS includes quadrantectomy with periareolar incision and glandular remodeling. The main indication for BCS was a complete clinical response to NACT or the presence of a neoplastic residue involving less than 20% of the glandular parenchyma. In BCS no treatment was performed on the contralateral breast. OPS included "inverted T mammoplasty", "J mammoplasty", "round block technique," and "batwing mammoplasty" and it forecast a reconstruction of the defect resulting from the removal of between 20% and 50% of the native breast tissue. The main indication for OPS was breast cancer with a non-optimal response after NAC, for which a standard conserving surgery with safe margins would either seem impossible or lead to a major deformity. In OPS, when indicated, we treated the healthy breast performing adjustment surgery. This included reduction mammoplasty or adjustment mastopexy.

CMR included nipple-sparing and skin-sparing mastectomy with breast prosthetic reconstruction and was indicated in patients with extensive or multicentric cancers or a tumor-to-breast volume ratio that required the excision of > 50% of the glandular volume, in case of inability to obtain clear surgical margins with OPS, of contraindications to adjuvant radiotherapy, or for patient preference.

Bilateral CMR was performed in patients with a bilateral breast tumor or in women with unilateral disease and a high risk of contralateral breast cancer, such as BRCA mutation carriers. In CMR, when indicated, we performed contralateral Mastopexy or breast augmentation.

Supplementary online references.

1. **P, Regnault.** Breast ptosis. Definition and treatment. *Clinics in Plastic Surgery* 1976;3(2):193-203.

2. **Franceschini G, Mason EJ, Grippo C, et al.** Image-Guided Localization Techniques for Surgical Excision of Non-Palpable Breast Lesions: An Overview of Current Literature and Our Experience with Preoperative Skin Tattoo. *J Pers Med* 2021;4;11(2):99. DOI: 10.3390/jpm11020099

3. **Rella R, Bufi E, Belli P, et al.** Magnetic Resonance Imaging prediction of large volume displacement oncoplastic surgery versus mastectomy in the treatment of breast cancer. *Ann Ital Chir* 2020;91:248-256.

4. **Cochrane RA, Valasiadou P, Wilson AR, Al-Ghazal SK, Macmillan RD.** Cosmesis and satisfaction after breast-conserving surgery correlates with the percentage of breast volume excised. *Br J Surg* 2003;90(12):1505-9. DOI: 10.1002/bjs.4344

5. **Katariya RN, Forrest AP, Gravelle IH.** Breast volumes in cancer of the breast. *Br J Cancer* 1974;29(3):270-273. DOI: 10.1038/bjc.1974.66

6. **Rancati AO, Angrigiani CH, Hammond DC, et al.** Direct to Implant Reconstruction in Nipple Sparing Mastectomy: Patient Selection by Preoperative Digital Mammogram. *Plast Reconstr Surg Glob Open* 2017;5(6):e1369. DOI: 10.1097/GOX.0000000000001369

7. **Breast Cancer (NCCN. Guidelines).** Clinical Practice Guidalines in Oncology. Versione 01.2022. *https://www.nccn.org/professionals/physician_gls/pdf/breast.pdf. 2022.* [Online] 2022.

8. ***Clinical Practice Guidalines in Oncology.*** *Breast Cancer (NCCN Guidelines). Versione 01.2022. Available at https://www.nccn.org/professionals/physician_gls/pdf/breast.pdf.* 2022.

Supplementary Figure:

Figure S1

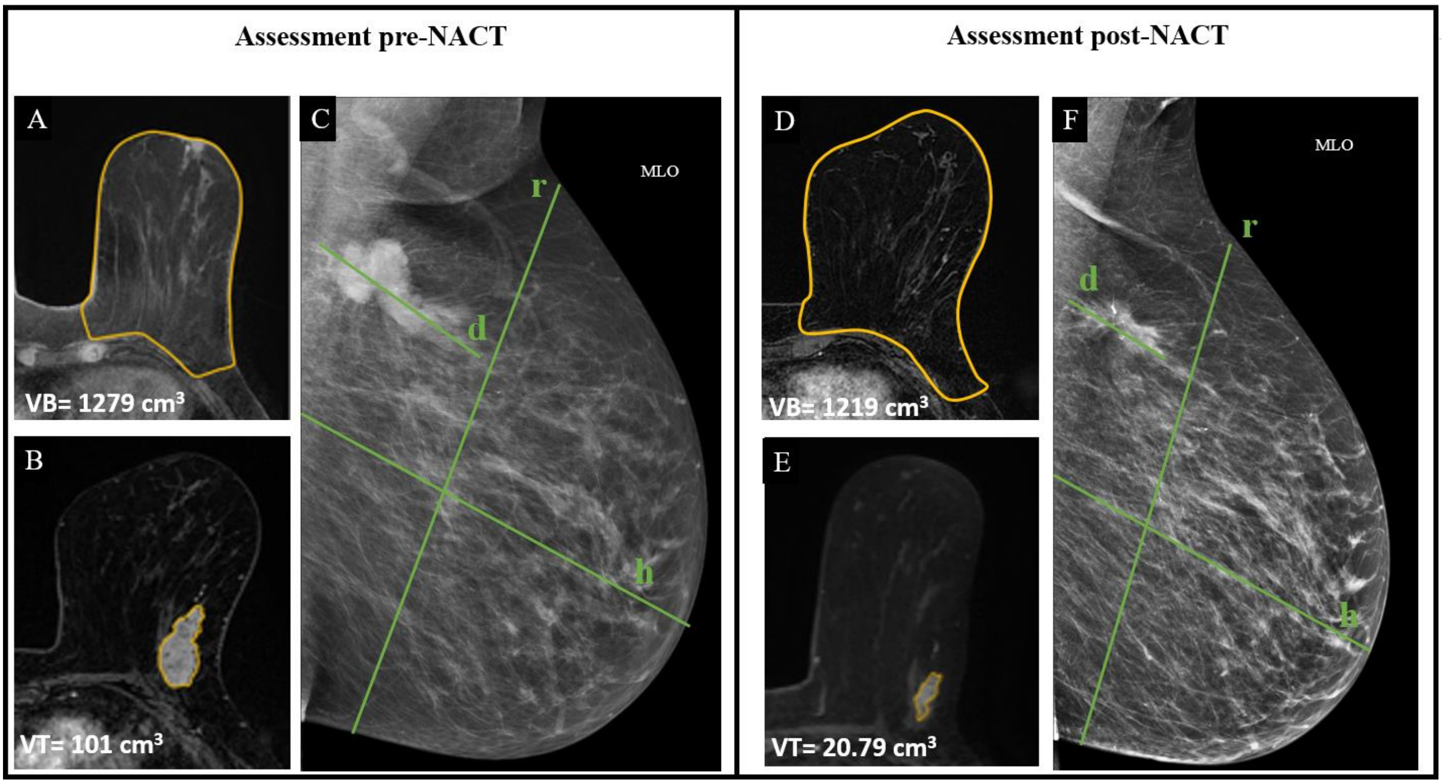

*Figure S1. **Definition of Prediction of the Excised Breast Area (PEBA).** The volume of the breast is calculated on the axial images with MRI (A. pre-NACT and D. post-NACT); the tumor volume was instead measured by surrounding the tumor with MRI (B. pre-NACT and E. post NACT). While in the evaluation of PEBA with RX, "r" describes the diameter of the mammary gland; "h" mammary gland height. "d" maximum length of the lesion. Vb= Volume of breast and Vt=volume of tumor (C. pre-NACT and F. post-NACT).*

Figure S2

Questionnaire 5. Thinking about the sensitivity of your breasts before surgery:											
Your breast has lost sensitivity?	Yes					No					
In case the answer is "Yes"											
How sensitive do you think you have lost?<br>0 = no loss; 100 =completely loss	0	10	20	30	40	50	60	70	80	90	100
How much has it affected your daily life?<br>0 = no influence; 100 =completely alterated	0	10	20	30	40	50	60	70	80	90	100
How much has it affected your sexual life?<br>0 = no influence; 100 =completely alterated	0	10	20	30	40	50	60	70	80	90	100

*Figure S2. **Evaluation of residual breast sensitivity and its influence on daily life** (Questionnaire 5).*

Figure S3

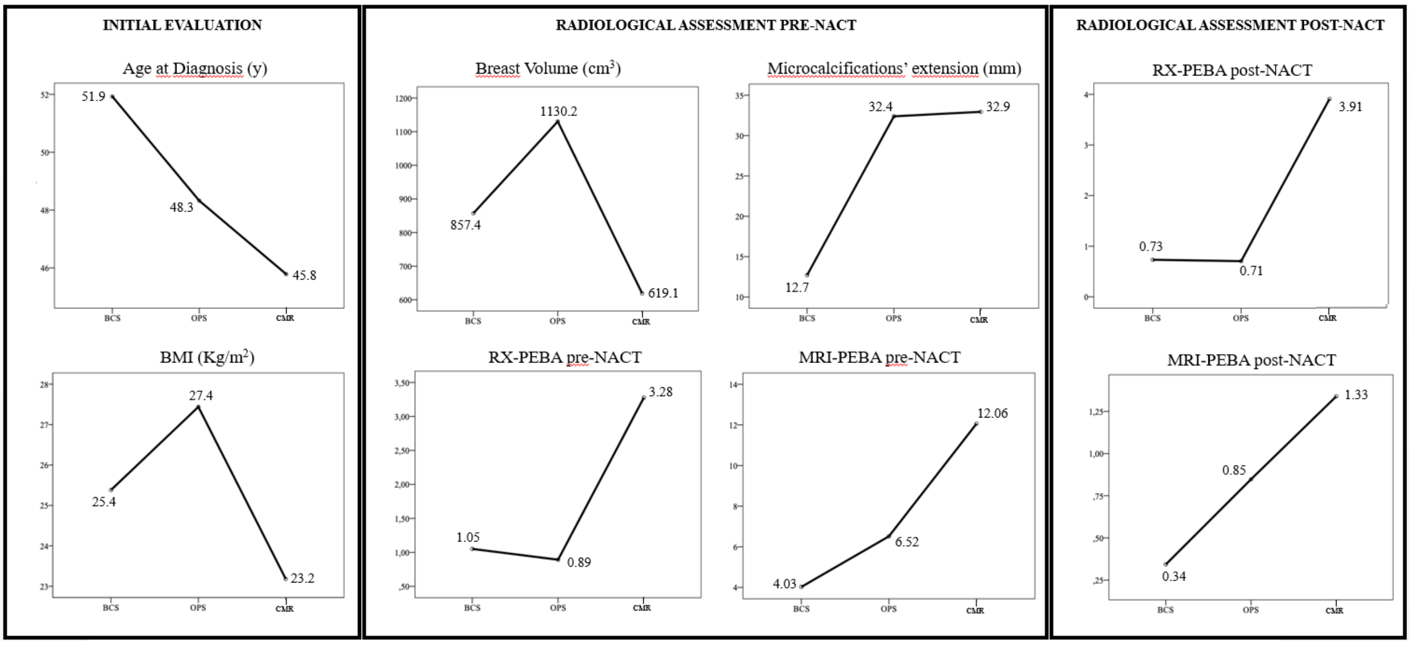

*Figure S3. **Average values obtained by type of surgery** in the initial patient evaluation (age and BMI); pre-NACT radiological evaluation (Breast volume calculated with MRI; extension of microcalcifications; RX-PEBA and MRI-PEBA) and post-NACT radiological assessment (RX-PEBA and MRI-PEBA). (BCS: breast conserving surgery; OPS: Oncoplastic Surgery; CMR: Conservative mastectomy with reconstruction; PEBA: Prevision of Excised Breast Area)*

Supplementary Table

Table S1

Table S1. Evaluation of pathological response

	BCS<br>118 (46.3%)	OPS<br>49 (19.2%)	CMR<br>88 (34.5%)	p-value
ypT				
- 0	43 (36.4%)	16 (32.7%)	25 (28.4%)	p=0.573
- ITC/mic	12 (10.2%)	6 (12.2%)	12 (13.6%)	
- 1	47 (39.8%)	17 (34.7%)	36 (40.8%)	

- 2	14 (11.9%)	6 (12.2%)	12 (13.6%)	
- 3	1 (0.8%)	4 (8.2%)	2 (2.3%)	
- 4	1 (0.8%)	0 (0%)	1 (1.1%)	
Histotype				
- DIC	57 (48.3%)	23 (46.9%)	45 (51.1%)	
- LIC	7 (5.9%)	2 (4.1%)	4 (4.5%)	p=0.719
- IC	11 (9.3%)	8 (16.3%)	14 (15.9%)	
- Not evaluable*	46 (36.4%)	16 (32.7%)	25 (28.4%)	
Ductal carcinoma in situ				
- Absence	85 (72%)	23 (46.9%)	57 (64.8%)	**p=0.009**
- Presence	33 (28%)	26 (51.3%)	31 (35.2%)	
Surgical specimen volume (cm3)	43.66 ± 34.4 (22.2 – 54.8)	120.63 ± 87.5 (55.0 – 165.9)	246.89 ± 195.5 (128 – 310.5)	**p<0.0001**
ypN				
- 0	69 (59%)	25 (51%)	42 (47.7%)	
- 0i+	5 (4.3%)	1 (2%)	7 (8%)	
- Mic	3 (2.6%)	2 (4.1%)	6 (6.8%)	p=0.446
- 1	27 (23.1%)	11 (22.4%)	17 (19.3%)	
- 2	11 (9.4%)	8 (16.3%)	11 (12.5%)	
- 3	2 (1.7%)	2 (4.1%)	5 (5.7%)	
Pathological complete response (pCR)				
- T	43 (36.4%)	33 (67.3%)	63 (71.6%)	p=0.471
- N	32 (27.1%)	15 (30.6%)	24 (27.3%)	p=0.900
- T and N	21 (17.8%)	7 (14.3%)	13 (14.8%)	p=0.833

* not evaluable for presence of ITC. micro residue of disease or pathological complete response. (BCS: breast conserving Surgery; OPS: level II oncoplastic surgery; CMR: conservative mastectomy with reconstruction)

Table S2

Table S2. Oncological radicality and oncological outcomes depending on type of surgery

	BCS 118 (46.3%)	OPS 49 (19.2%)	CMR 88 (34.5%)	p-value
Oncological radicality				
- Reached	105 (89%)	48 (98%)	80 (90.9%)	p=0.171
- Failed*	13 (11%)	1 (2%)	8 (9.1%)	
Oncological outcomes				
N. cases of loco-regional relapse				
- Breast	3 (2.5%)	1 (2%)	3 (3.4%)	p=0.908
- Axilla	1 (0.8%)	1 (2%)	1 (1.1%)	
N. cases of systemic relapse	12 (10.2%)	5 (10.2%)	12 (13.2%)	p=0.734
N. of deaths	6 (5.1%)	3 (6.1%)	6 (6.8%)	p=0.833

* presence of margin infiltration by the invasive cancer or DCIS or presence of either on surgical cavity shavings. BCS: breast conserving surgery; OPS: Level II oncoplastic surgery; CMR: conservative mastectomy with reconstruction

Table S3

Table S3. Univariate and multivariable analysis for BCS

	Univariate Analysis	Multivariable Analysis
Age (y) - > 45.6 [vs < 45.5]	p<0.0001; OR 3.025	
BMI - Between 21.17 and 24.22 [vs < 21.16 o > 24.23]	p=0.025; OR 0. 540	
Pathological mutation of BRCA 1 and 2 [vs <no pathogenetic variant]	p=0.345; OR 0.275	

Bra size<br>- 3<br>[vs ≤ 2 o ≥ 4]	p=0.039; OR 1.722	
Rancati score<br>- 1<br>- 2<br>- 3	p=0.166; OR 0.682<br>p=0.979; OR 1.039<br>p=0.124; OR 1.709	
Microcalcification extension<br>- < 21.9 mm<br>[vs > 22 mm]	p<0.0001; OR 3.888	
N. involved quadrants<br>- 1<br>[vs ≥ 2]	p<0.0001; OR 3.715	
Grade of ptosis<br>- 1<br>[vs 2; 3]	p=0.019; OR 1.844	**β 0.511; p=0.098; OR 1.667<br>(95% CI -0.178 – 1.272)**
Breast volume evaluated with MRI<br>- Between 646.00 and 1009.39 cm3<br>[vs <655.9 o > 1009.4 cm3]	p=0.010; OR 1.977	**β 0.900; p=0.012; OR 2.460<br>(95% CI 0.182 – 1.735)**
Focality/centricity pre NACT<br>- Unifocal<br>[vs multifocal o multicentric]	P<0.0001; OR 5.991	**β 1.368; p<0.0001; OR 3.927<br>(95% CI 0.657 – 2.172)**
RX-PEBA pre NACT<br>- <0.44<br>[vs > 0.45]	P<0.0001; OR 4.576	
MRI-PEBA pre NACT<br>- < 3.52<br>[vs > 3.53]	P<0.0001; OR 6.263	**β 1.251; p=0.001; OR 3.495<br>(95% CI 0.558 – 2.023)**
Focality/centricity post NACT<br>- Clinical complete response<br>- Unifocal<br>[vs multifocal o multicentric]	P<0.0001; OR 6.137	**β 1.536; p=0.001; OR 4.646<br>(95% CI 0.673 – 2.615)**
RX-PEBA post NACT<br>- < 0.041<br>[vs > 0.042]	P<0.0001; OR 5.906	**β 1.505; p=0.001; OR 4.502<br>(95% CI 0.803 – 2.298)**
MRI-PEBA post NACT<br>- < 0.26<br>[vs > 0.27]	P<0.0001; OR 2.764	

Table S4

Table S4. Univariate and multivariable analysis for OPS		
	Univariate Analysis	Multivariable Analysis
Age (y)<br>- 43 - 45.59<br>[vs < 42.9 o >45.6]	p=0.031; OR 2.524	
BMI<br>- > 24.23<br>[vs < 24.22]	p=0.015; OR 2.220	
BRCA 1 and 2 pathological mutation<br>[vs <no pathogenetic variant]	p=0.082; OR 0.166	
Bra size<br>- ≥ 4<br>[vs ≤ 3]	p<0.0001; OR 3.169	
Rancati score<br>- 1<br>[vs grade 2 or 3]	p=0.019; OR 0.379	
Microcalcification extension	p=0.004; OR 2.566	

- 22 – 79.9 mm<br>[vs < 21.9 o > 80 mm]		
N. involved quadrants<br>- 2<br>[vs 1 o ≥ 3 quadrants]	p=0.203; OR 1.553	
Ptosis<br>- 2 – 3<br>[vs grade 0 or 1]	p<0.0001; OR 4.191	**β 1.352; p=0.002; OR 3.866<br>(95% CI 0.466 – 2.657)**
Breast volume evaluated with MRI<br>- >1009.4 cm3<br>[vs < 1009.3 cm3]	p<0.0001; OR 5.054	**β 1.526; p=0.002; OR 4.598<br>(95% CI 0.559 – 2.766)**
Focality/centricity pre NACT<br>- Multifocality<br>[vs < unifocality or multicentricity]	P<0.0001; OR 8.345	**β 1.193; p=0.012; OR 3.297<br>(95% CI 0.180 – 2.373)**
RX-PEBA pre NACT<br>- 0.45 - 1.35<br>[vs < 0.44 o > 1.36]	p=0.049; OR 0.505	
MRI-PEBA pre NACT<br>- 3.53 – 9.99<br>[vs < 3.52 o > 10.0]	p<0.0001; OR 4.186	**β 1.391; p=0.006; OR 4.021<br>(95% CI 0.399 – 2.606)**
Focality/centricity post NACT<br>- Multifocality<br>[vs <clinical complete response or. unifocality or multicentricity]	p<0.0001; OR 8.782	**β 2.274; p=0.001; OR 9.714<br>(95% CI 1.190 – 3.784)**
RX-PEBA post NACT<br>- 0.042 – 4.61<br>[vs < 0.041 o > 4.62]	p<0.0001; OR 5.909	**β 2.020; p=0.001; OR 7.553<br>(95% CI 1.117 – 3.391)**
MRI-PEBA post NACT<br>- 0.27 – 1.29<br>[vs < 0.26 o > 1.30]	p=0.001; OR 2.843	

Table S5

Table S5. Univariate and multivariable analysis for CMR		
	Univariate A.	Multivariate A.
Age (y)<br>< 42.9<br>[vs > 43]	p=0.001; OR 2.612	
BMI<br>< 21.16<br>[vs > 21.17]	p=0.002; OR 2.637	
BRCA 1 and 2 pathological mutation<br>[vs <no pathogenetic variant]	p<0.0001; OR 6.900	**β 2.867; p=0.010; OR 17.581<br>(95% CI 1.263 – 4.748)**
Bra size<br>1 – 2<br>[vs ≥ 3]	p<0.0001; OR 3.143	
Rancati score<br>3<br>[vs score 1 or 2]	p=0.007; OR 0.286	
Microcalcification extension<br>> 80 mm<br>[vs <79.9 mm]	p=0.044; OR 1.736	
N. involved quadrants<br>3 or more quadrants<br>[vs ≤ 2 quadrants]	p<0.0001; OR 4.731	
Ptosis<br>0<br>[vs grade 1; 2; 3]	p<0.0001; OR 6.261	**β 1.389; p=0.009; OR 4.010<br>(95% CI 0.303 – 2.727)**

Breast volume evaluated with MRI < 645.99 cm3 [vs > 656 cm3]	p<0.0001; OR 5.141	**β 1.375; p=0.004; OR 3.954 (95% CI 0.414 – 2.595)**
Focality/centricity pre NACT Multicentricity [vs < uni- e multifocality]	P<0.0001; OR 15.002	**β 2.309; p=0.001; OR 10.068 (95% CI 1.301 – 3.858)**
RX-PEBA pre NACT > 1.36 [vs <1.35]	p<0.0001; OR 13.632	
MRI-PEBA pre NACT > 10.00 [vs <9.99]	p<0.0001; OR 10.281	**β 1.860; p=0.001; OR 6.426 (95% CI 0.669 – 3.448)**
Focality/centricity post NACT Multicentricity [vs <clinical complete response or. unifocality or multifocality]	p<0.0001; OR 16.171	**β 2.007; p=0.005; OR 7.440 (95% CI 0.655 – 20.651)**
RX-PEBA post NACT > 4.62 [vs <4.61]	p<0.0001; OR 12.772	**β 1.598; p=0.020; OR 4.943 (95% CI 0.202 – 3.975)**
MRI-PEBA post NACT > 1.30 [vs <1.29]	p<0.0001; OR 4.967	
